# Ethyl 3-(4-chloro­phen­yl)-1-(2-oxo-2-phenyl­eth­yl)-1*H*-pyrazole-5-carboxyl­ate

**DOI:** 10.1107/S1600536811025918

**Published:** 2011-07-06

**Authors:** Liang-Wen Zheng, Yin-Rui Liu, Bao-Xiang Zhao

**Affiliations:** a School of Chemistry and Chemical Engineering, Shandong University, Jinan 250100, People’s Republic of China

## Abstract

In the title compound, C_20_H_17_ClN_2_O_3_, the dihedral angles between the pyrazole ring and the substituted and unsubstituted benzene rings are 3.64 (13) and 81.15 (17)°, respectively. Mol­ecules are connected *via* three pairs of weak hydrogen bonds into a centrosymmetric dimer. The crystal structure is stabilized by inter­molecular C—H⋯O and C—H⋯π inter­actions.

## Related literature

For applications of pyrazoles, see: Kosuge & Kamiya (1962 ▶); Ganesan (1996 ▶); Farag *et al.* (2010 ▶); Boschi *et al.* (2011 ▶); Kasımoğullan *et al.* (2010 ▶); Christodoulou *et al.* (2010 ▶); Scanio *et al.* (2010 ▶); Da Sliva *et al.* (2010 ▶). For related structures, see: Xie *et al.* (2009 ▶); Arban *et al.* (2010 ▶). For the synthesis of ethyl 3-(4-chloro­phen­yl)-1-(2-oxo-2-phenyl­eth­yl)-1*H*-pyrazole-5-carboxyl­ate, see: Zheng *et al.* (2010 ▶). 
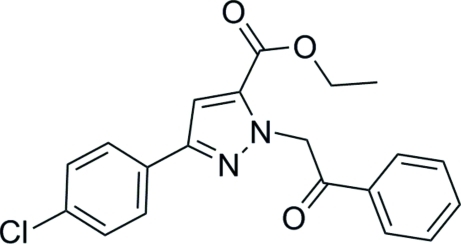


## Experimental

### 

#### Crystal data

C_20_H_17_ClN_2_O_3_
*M* *_r_* = 368.81
Triclinic, 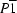

*a* = 7.7238 (10) Å
*b* = 8.382 (1) Å
*c* = 15.8143 (18) Å
α = 98.667 (2)°
β = 93.828 (2)°
γ = 113.849 (2)°
*V* = 916.44 (19) Å^3^
*Z* = 2
Mo *K*α radiation
μ = 0.23 mm^−1^
*T* = 296 K
0.12 × 0.10 × 0.06 mm

#### Data collection

Bruker APEXII CCD area-detector diffractometer
Absorption correction: multi-scan (*SADABS*; Bruker, 2007 ▶) *T*_min_ = 0.973, *T*_max_ = 0.986
4894 measured reflections
3216 independent reflections
2079 reflections with *I* > 2σ(*I*)
*R*_int_ = 0.018

#### Refinement

*R*[*F* ^2^ > 2σ(*F* ^2^)] = 0.048
*wR*(*F* ^2^) = 0.123
*S* = 1.04
3216 reflections
237 parameters
H-atom parameters constrained
Δρ_max_ = 0.17 e Å^−3^
Δρ_min_ = −0.18 e Å^−3^

### 

Data collection: *APEX2* (Bruker, 2007 ▶); cell refinement: *SAINT* (Bruker, 2007 ▶); data reduction: *SAINT*; program(s) used to solve structure: *SHELXS97* (Sheldrick, 2008 ▶); program(s) used to refine structure: *SHELXL97* (Sheldrick, 2008 ▶); molecular graphics: *ORTEP-3* (Farrugia, 1997 ▶); software used to prepare material for publication: *SHELXTL* (Sheldrick, 2008 ▶).

## Supplementary Material

Crystal structure: contains datablock(s) I, global. DOI: 10.1107/S1600536811025918/vm2105sup1.cif

Structure factors: contains datablock(s) I. DOI: 10.1107/S1600536811025918/vm2105Isup2.hkl

Supplementary material file. DOI: 10.1107/S1600536811025918/vm2105Isup3.cml

Additional supplementary materials:  crystallographic information; 3D view; checkCIF report

## Figures and Tables

**Table 1:** Hydrogen-bond geometry (Å, °) *Cg*1 is the centroid of the C7–C12 ring.

*D*—H⋯*A*	*D*—H	H⋯*A*	*D*⋯*A*	*D*—H⋯*A*
C5—H5⋯O1^i^	0.93	2.52	3.410 (3)	161
C8—H8⋯O1^i^	0.93	2.42	3.348 (4)	180
C9—H9⋯O3^ii^	0.93	2.56	3.434 (3)	157
C13—H13*A*⋯*Cg*1^iii^	0.97	2.80	3.605 (3)	140

Symmetry codes: (i) 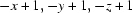; (ii) 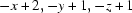; (iii) 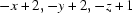.

**Fig. 1.**
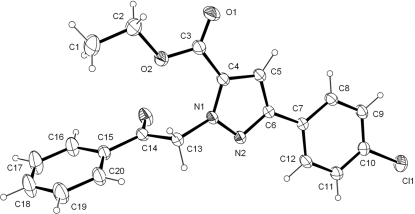
ORTEP view of compound (I), showing 25% probability displacement ellipsoids.

**Fig. 2.**
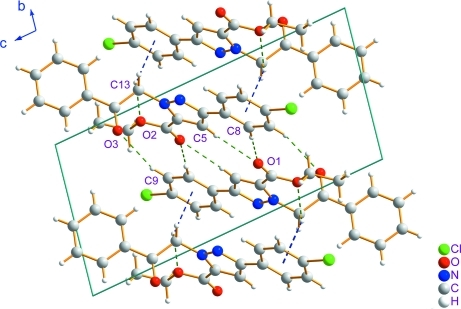
Packing diagram of compound (I). Short contacts and C—H···π are shown as dashed lines.

## supplementary crystallographic information

### Comment

Pyrazoles form an important class of analogues, which occupy a special role in natural and synthetic compounds (Kosuge *et al.*, 1962; Ganesan *et al.*, 1996). Pyrazole derivatives have been the subject of much research because of their importance in various applications and their widespread potential biological and pharmacological activities, such as antitumor (Farag *et al.*, 2010), antimicrobial (Boschi *et al.*, 2011), antiglaucoma (Kasımoğullan *et al.*, 2010), anti-angiogenic (Christodoulou *et al.*, 2010), antinociceptive (Scanio *et al.*, 2010) and anti-inflammatory (Da Sliva *et al.*, 2010).

One chlorine substituted benzene group, an ethyl carboxylate moiety and a 2-oxo-2-phenylethyl moiety are bonded to core pyrazole ring in the title molecule at C6, C4, N1 as showed in Fig. 1. Torsion angle N1—C4—C3—O1 of 175.6 (2)° illustrates that O1 in the ethyl carboxylate moiety adopts a antiperiplanar conformation with respect to the N1 atom of the pyrazole ring. The pyrazole core structure and the chlorine substituted benzene are approximately coplanar with a dihedral angle of 3.65 (7)°. A S(6) pseudo-ring closed by a C13—H13A···O2 intramolecular interaction is observed. The carbonyl O1 atom of the ethyl formate moiety interacts with the pyrazole H atom of the adjacent molecules through a pair of linear C5—H5···O1 hydrogen bonds to form a ten-membered ring motif. Moreover, some other C–H···O and C—H···π interactions (Table 1) may supply further coulombic stabilization and take part in formation of the three-dimensional structure.

### Experimental

To dried acetonitrile (50 ml), ethyl 3-(4-chlorophenyl)-1*H*-pyrazole-5-carboxylate (2.50 g, 10 mmol), 2-bromo-1-phenylethanone (2.00 g, 10 mmol) and potassium carbonate (2.76 g, 20 mmol) were added. The mixture was heated to reflux for 1 h, until TLC indicated the end of reaction. The reaction mixture was cooled to room temperature and filtered. The filtrate was concentrated under reduced pressure and the residue was purified by column chromatography using ethyl acetate/petroleum ether (*v*/*v* = 1:4) as eluant to afford compound ethyl 3-(4-chlorophenyl)-1-(2-oxo-2-phenylethyl)-1*H*-pyrazole-5-carboxylate (I) in 70% yield. Crystals of (I) suitable for X-ray diffraction were obtained by slow evaporation of a solution of the solid in ethyl acetate at room temperature for 7 days.

### Refinement

All H atoms attached to C atoms were placed in their calculated positions (methyl C–H = 0.96 Å, methylene C–H = 0.97 Å and aromatic C–H = 0.93 Å) and were refined using a riding model with *U*iso(H) = 1.5*U*eq for methyl groups and with *U*iso(H) = 1.2*U*eq for others.

### Crystal data

**Table:**

C_20_H_17_ClN_2_O_3_	*Z* = 2
*M**_r_* = 368.81	*F*(000) = 384
Triclinic, *P*1	*D*_x_ = 1.337 Mg m^−^^3^
Hall symbol: -P 1	Mo *K*α radiation, λ = 0.71073 Å
*a* = 7.7238 (10) Å	Cell parameters from 1101 reflections
*b* = 8.382 (1) Å	θ = 2.7–22.0°
*c* = 15.8143 (18) Å	µ = 0.23 mm^−^^1^
α = 98.667 (2)°	*T* = 296 K
β = 93.828 (2)°	Block, colorless
γ = 113.849 (2)°	0.12 × 0.10 × 0.06 mm
*V* = 916.44 (19) Å^3^	

### Data collection

**Table:**

Bruker APEXII CCD area-detector diffractometer	3216 independent reflections
Radiation source: fine-focus sealed tube	2079 reflections with *I* > 2σ(*I*)
graphite	*R*_int_ = 0.018
phi and ω scans	θ_max_ = 25.1°, θ_min_ = 2.6°
Absorption correction: multi-scan (*SADABS*; Bruker, 2007)	*h* = −9→8
*T*_min_ = 0.973, *T*_max_ = 0.986	*k* = −9→9
4894 measured reflections	*l* = −18→10

### Refinement

**Table:**

Refinement on *F*^2^	Secondary atom site location: difference Fourier map
Least-squares matrix: full	Hydrogen site location: inferred from neighbouring sites
*R*[*F*^2^ > 2σ(*F*^2^)] = 0.048	H-atom parameters constrained
*wR*(*F*^2^) = 0.123	*w* = 1/[σ^2^(*F*_o_^2^) + (0.0468*P*)^2^ + 0.1653*P*] where *P* = (*F*_o_^2^ + 2*F*_c_^2^)/3
*S* = 1.04	(Δ/σ)_max_ < 0.001
3216 reflections	Δρ_max_ = 0.17 e Å^−^^3^
237 parameters	Δρ_min_ = −0.18 e Å^−^^3^
0 restraints	Extinction correction: *SHELXL97* (Sheldrick, 2008), Fc^*^=kFc[1+0.001xFc^2^λ^3^/sin(2θ)]^-1/4^
Primary atom site location: structure-invariant direct methods	Extinction coefficient: 0.008 (2)

### Special details

**Table:**

Geometry. All e.s.d.'s (except the e.s.d. in the dihedral angle between two l.s. planes) are estimated using the full covariance matrix. The cell e.s.d.'s are taken into account individually in the estimation of e.s.d.'s in distances, angles and torsion angles; correlations between e.s.d.'s in cell parameters are only used when they are defined by crystal symmetry. An approximate (isotropic) treatment of cell e.s.d.'s is used for estimating e.s.d.'s involving l.s. planes.
Refinement. Refinement of *F*^2^ against ALL reflections. The weighted *R*-factor *wR* and goodness of fit *S* are based on *F*^2^, conventional *R*-factors *R* are based on *F*, with *F* set to zero for negative *F*^2^. The threshold expression of *F*^2^ > σ(*F*^2^) is used only for calculating *R*-factors(gt) *etc*. and is not relevant to the choice of reflections for refinement. *R*-factors based on *F*^2^ are statistically about twice as large as those based on *F*, and *R*- factors based on ALL data will be even larger.

### Fractional atomic coordinates and isotropic or equivalent isotropic displacement parameters (Å^2^)

**Table:**

	*x*	*y*	*z*	*U*_iso_*/*U*_eq_	
Cl1	1.39705 (12)	0.55340 (11)	0.24320 (5)	0.0857 (3)	
O1	0.4703 (3)	0.6797 (3)	0.61211 (13)	0.0882 (7)	
O2	0.6629 (2)	0.8989 (2)	0.71892 (11)	0.0689 (5)	
O3	1.0787 (3)	0.9089 (2)	0.79687 (11)	0.0765 (6)	
N1	0.9783 (3)	0.9370 (2)	0.63117 (12)	0.0502 (5)	
N2	1.1032 (3)	0.9166 (2)	0.58057 (12)	0.0516 (5)	
C1	0.5722 (5)	1.0065 (5)	0.8478 (2)	0.1137 (13)	
H1A	0.6625	0.9790	0.8802	0.171*	
H1B	0.4675	0.9957	0.8795	0.171*	
H1C	0.6337	1.1260	0.8382	0.171*	
C2	0.4997 (4)	0.8818 (4)	0.7639 (2)	0.0894 (10)	
H2A	0.4340	0.7609	0.7728	0.107*	
H2B	0.4103	0.9101	0.7300	0.107*	
C3	0.6271 (4)	0.7888 (3)	0.64366 (16)	0.0583 (7)	
C4	0.7978 (3)	0.8102 (3)	0.60278 (14)	0.0490 (6)	
C5	0.8081 (3)	0.7040 (3)	0.53051 (15)	0.0510 (6)	
H5	0.7078	0.6056	0.4964	0.061*	
C6	0.9993 (3)	0.7733 (3)	0.51840 (14)	0.0470 (6)	
C7	1.0940 (3)	0.7141 (3)	0.45134 (14)	0.0474 (6)	
C8	0.9962 (4)	0.5635 (3)	0.38749 (15)	0.0532 (6)	
H8	0.8667	0.4953	0.3875	0.064*	
C9	1.0887 (4)	0.5136 (3)	0.32388 (15)	0.0571 (7)	
H9	1.0221	0.4119	0.2816	0.069*	
C10	1.2795 (4)	0.6148 (3)	0.32337 (16)	0.0591 (7)	
C11	1.3793 (4)	0.7624 (4)	0.38583 (17)	0.0725 (8)	
H11	1.5090	0.8297	0.3856	0.087*	
C12	1.2863 (4)	0.8106 (4)	0.44906 (17)	0.0697 (8)	
H12	1.3550	0.9113	0.4916	0.084*	
C13	1.0477 (3)	1.0912 (3)	0.70068 (14)	0.0530 (6)	
H13A	0.9535	1.1403	0.7030	0.064*	
H13B	1.1641	1.1808	0.6876	0.064*	
C14	1.0880 (3)	1.0536 (3)	0.78837 (15)	0.0530 (6)	
C15	1.1408 (3)	1.2016 (3)	0.86377 (16)	0.0556 (7)	
C16	1.1626 (4)	1.3698 (4)	0.85486 (18)	0.0757 (9)	
H16	1.1464	1.3938	0.8001	0.091*	
C17	1.2085 (5)	1.5024 (4)	0.9269 (2)	0.1021 (12)	
H17	1.2223	1.6153	0.9205	0.122*	
C18	1.2337 (6)	1.4687 (5)	1.0071 (2)	0.1132 (13)	
H18	1.2645	1.5583	1.0555	0.136*	
C19	1.2139 (6)	1.3030 (5)	1.0166 (2)	0.1131 (13)	
H19	1.2313	1.2801	1.0715	0.136*	
C20	1.1684 (5)	1.1703 (4)	0.94532 (18)	0.0823 (9)	
H20	1.1561	1.0582	0.9523	0.099*	

### Atomic displacement parameters (Å^2^)

**Table:**

	*U*^11^	*U*^22^	*U*^33^	*U*^12^	*U*^13^	*U*^23^
Cl1	0.0942 (6)	0.0923 (6)	0.0644 (5)	0.0382 (5)	0.0278 (4)	−0.0068 (4)
O1	0.0441 (11)	0.1021 (15)	0.0846 (15)	0.0121 (11)	0.0016 (10)	−0.0234 (12)
O2	0.0548 (11)	0.0744 (12)	0.0650 (12)	0.0223 (9)	0.0134 (9)	−0.0112 (10)
O3	0.1026 (16)	0.0619 (12)	0.0619 (13)	0.0395 (11)	−0.0061 (10)	−0.0036 (9)
N1	0.0477 (12)	0.0485 (11)	0.0419 (11)	0.0137 (10)	0.0032 (9)	−0.0072 (9)
N2	0.0477 (12)	0.0529 (12)	0.0432 (12)	0.0146 (10)	0.0045 (9)	−0.0031 (9)
C1	0.105 (3)	0.139 (3)	0.091 (3)	0.057 (3)	0.031 (2)	−0.017 (2)
C2	0.064 (2)	0.111 (3)	0.086 (2)	0.0386 (18)	0.0236 (17)	−0.0116 (19)
C3	0.0511 (17)	0.0620 (16)	0.0549 (17)	0.0217 (14)	0.0052 (13)	−0.0024 (13)
C4	0.0445 (14)	0.0495 (14)	0.0456 (14)	0.0162 (11)	0.0006 (11)	0.0006 (11)
C5	0.0455 (14)	0.0508 (14)	0.0458 (14)	0.0141 (11)	0.0000 (11)	−0.0020 (11)
C6	0.0460 (14)	0.0478 (13)	0.0395 (13)	0.0148 (11)	0.0011 (11)	0.0025 (11)
C7	0.0476 (14)	0.0473 (13)	0.0397 (13)	0.0152 (11)	0.0025 (10)	0.0016 (10)
C8	0.0516 (15)	0.0459 (13)	0.0523 (15)	0.0147 (12)	0.0016 (12)	0.0004 (11)
C9	0.0685 (18)	0.0459 (14)	0.0469 (15)	0.0202 (13)	−0.0006 (12)	−0.0052 (11)
C10	0.0642 (18)	0.0605 (16)	0.0469 (15)	0.0238 (14)	0.0112 (12)	−0.0006 (12)
C11	0.0555 (17)	0.0782 (19)	0.0575 (17)	0.0099 (14)	0.0136 (13)	−0.0126 (14)
C12	0.0553 (17)	0.0687 (17)	0.0556 (17)	0.0071 (14)	0.0071 (13)	−0.0184 (13)
C13	0.0524 (15)	0.0494 (14)	0.0453 (15)	0.0143 (12)	0.0044 (11)	−0.0038 (11)
C14	0.0453 (15)	0.0537 (15)	0.0508 (16)	0.0166 (12)	0.0034 (11)	−0.0033 (12)
C15	0.0529 (16)	0.0596 (16)	0.0472 (16)	0.0225 (13)	0.0028 (12)	−0.0047 (12)
C16	0.096 (2)	0.0646 (18)	0.0563 (18)	0.0314 (16)	0.0044 (15)	−0.0087 (14)
C17	0.146 (3)	0.069 (2)	0.077 (3)	0.042 (2)	0.009 (2)	−0.0134 (18)
C18	0.161 (4)	0.097 (3)	0.065 (2)	0.055 (3)	0.002 (2)	−0.028 (2)
C19	0.167 (4)	0.112 (3)	0.052 (2)	0.063 (3)	−0.005 (2)	−0.010 (2)
C20	0.111 (3)	0.080 (2)	0.0514 (18)	0.0417 (19)	0.0006 (16)	−0.0029 (15)

### Geometric parameters (Å, °)

**Table:**

Cl1—C10	1.741 (2)	C8—H8	0.9300
O1—C3	1.199 (3)	C9—C10	1.373 (3)
O2—C3	1.328 (3)	C9—H9	0.9300
O2—C2	1.456 (3)	C10—C11	1.363 (3)
O3—C14	1.214 (3)	C11—C12	1.374 (3)
N1—N2	1.340 (2)	C11—H11	0.9300
N1—C4	1.360 (3)	C12—H12	0.9300
N1—C13	1.449 (3)	C13—C14	1.508 (3)
N2—C6	1.346 (3)	C13—H13A	0.9700
C1—C2	1.475 (4)	C13—H13B	0.9700
C1—H1A	0.9600	C14—C15	1.486 (3)
C1—H1B	0.9600	C15—C20	1.374 (4)
C1—H1C	0.9600	C15—C16	1.381 (4)
C2—H2A	0.9700	C16—C17	1.380 (4)
C2—H2B	0.9700	C16—H16	0.9300
C3—C4	1.465 (3)	C17—C18	1.358 (5)
C4—C5	1.367 (3)	C17—H17	0.9300
C5—C6	1.390 (3)	C18—C19	1.368 (5)
C5—H5	0.9300	C18—H18	0.9300
C6—C7	1.464 (3)	C19—C20	1.373 (4)
C7—C12	1.381 (3)	C19—H19	0.9300
C7—C8	1.388 (3)	C20—H20	0.9300
C8—C9	1.381 (3)		
			
C3—O2—C2	116.6 (2)	C8—C9—H9	120.2
N2—N1—C4	111.60 (17)	C11—C10—C9	120.7 (2)
N2—N1—C13	117.93 (18)	C11—C10—Cl1	119.4 (2)
C4—N1—C13	130.3 (2)	C9—C10—Cl1	119.94 (19)
N1—N2—C6	105.50 (18)	C10—C11—C12	119.3 (2)
C2—C1—H1A	109.5	C10—C11—H11	120.4
C2—C1—H1B	109.5	C12—C11—H11	120.4
H1A—C1—H1B	109.5	C11—C12—C7	122.0 (2)
C2—C1—H1C	109.5	C11—C12—H12	119.0
H1A—C1—H1C	109.5	C7—C12—H12	119.0
H1B—C1—H1C	109.5	N1—C13—C14	114.3 (2)
O2—C2—C1	107.8 (2)	N1—C13—H13A	108.7
O2—C2—H2A	110.2	C14—C13—H13A	108.7
C1—C2—H2A	110.2	N1—C13—H13B	108.7
O2—C2—H2B	110.2	C14—C13—H13B	108.7
C1—C2—H2B	110.2	H13A—C13—H13B	107.6
H2A—C2—H2B	108.5	O3—C14—C15	121.5 (2)
O1—C3—O2	123.5 (2)	O3—C14—C13	121.4 (2)
O1—C3—C4	122.6 (2)	C15—C14—C13	117.1 (2)
O2—C3—C4	113.8 (2)	C20—C15—C16	118.7 (2)
N1—C4—C5	106.7 (2)	C20—C15—C14	119.0 (2)
N1—C4—C3	126.4 (2)	C16—C15—C14	122.3 (2)
C5—C4—C3	126.8 (2)	C17—C16—C15	120.3 (3)
C4—C5—C6	105.9 (2)	C17—C16—H16	119.9
C4—C5—H5	127.1	C15—C16—H16	119.9
C6—C5—H5	127.1	C18—C17—C16	120.2 (3)
N2—C6—C5	110.3 (2)	C18—C17—H17	119.9
N2—C6—C7	119.5 (2)	C16—C17—H17	119.9
C5—C6—C7	130.1 (2)	C17—C18—C19	120.0 (3)
C12—C7—C8	117.6 (2)	C17—C18—H18	120.0
C12—C7—C6	120.2 (2)	C19—C18—H18	120.0
C8—C7—C6	122.3 (2)	C18—C19—C20	120.2 (3)
C9—C8—C7	120.8 (2)	C18—C19—H19	119.9
C9—C8—H8	119.6	C20—C19—H19	119.9
C7—C8—H8	119.6	C19—C20—C15	120.7 (3)
C10—C9—C8	119.6 (2)	C19—C20—H20	119.7
C10—C9—H9	120.2	C15—C20—H20	119.7
			
C4—N1—N2—C6	0.1 (3)	C7—C8—C9—C10	−0.5 (4)
C13—N1—N2—C6	−175.2 (2)	C8—C9—C10—C11	1.1 (4)
C3—O2—C2—C1	176.2 (3)	C8—C9—C10—Cl1	−179.8 (2)
C2—O2—C3—O1	0.3 (4)	C9—C10—C11—C12	−0.9 (5)
C2—O2—C3—C4	−178.7 (2)	Cl1—C10—C11—C12	−180.0 (2)
N2—N1—C4—C5	−0.1 (3)	C10—C11—C12—C7	−0.1 (5)
C13—N1—C4—C5	174.5 (2)	C8—C7—C12—C11	0.7 (4)
N2—N1—C4—C3	178.1 (2)	C6—C7—C12—C11	−178.2 (3)
C13—N1—C4—C3	−7.4 (4)	N2—N1—C13—C14	−100.5 (2)
O1—C3—C4—N1	175.6 (3)	C4—N1—C13—C14	85.2 (3)
O2—C3—C4—N1	−5.4 (4)	N1—C13—C14—O3	6.4 (3)
O1—C3—C4—C5	−6.6 (4)	N1—C13—C14—C15	−174.04 (19)
O2—C3—C4—C5	172.4 (2)	O3—C14—C15—C20	−4.0 (4)
N1—C4—C5—C6	0.1 (3)	C13—C14—C15—C20	176.4 (2)
C3—C4—C5—C6	−178.1 (2)	O3—C14—C15—C16	176.2 (3)
N1—N2—C6—C5	0.0 (3)	C13—C14—C15—C16	−3.4 (4)
N1—N2—C6—C7	179.9 (2)	C20—C15—C16—C17	−0.9 (5)
C4—C5—C6—N2	0.0 (3)	C14—C15—C16—C17	178.8 (3)
C4—C5—C6—C7	−179.9 (2)	C15—C16—C17—C18	0.4 (5)
N2—C6—C7—C12	−3.7 (4)	C16—C17—C18—C19	0.1 (6)
C5—C6—C7—C12	176.1 (3)	C17—C18—C19—C20	0.0 (7)
N2—C6—C7—C8	177.5 (2)	C18—C19—C20—C15	−0.5 (6)
C5—C6—C7—C8	−2.7 (4)	C16—C15—C20—C19	1.0 (5)
C12—C7—C8—C9	−0.4 (4)	C14—C15—C20—C19	−178.8 (3)
C6—C7—C8—C9	178.4 (2)		

### Hydrogen-bond geometry (Å, °)

**Table:**

Cg1 is the centroid of the C7–C12 ring.

**Table:**

*D*—H···*A*	*D*—H	H···*A*	*D*···*A*	*D*—H···*A*
C5—H5···O1^i^	0.93	2.52	3.410 (3)	161
C8—H8···O1^i^	0.93	2.42	3.348 (4)	180
C9—H9···O3^ii^	0.93	2.56	3.434 (3)	157
C13—H13A···Cg1^iii^	0.97	2.80	3.605 (3)	140

Symmetry codes: (i) −*x*+1, −*y*+1, −*z*+1; (ii) −*x*+2, −*y*+1, −*z*+1; (iii) −*x*+2, −*y*+2, −*z*+1.
